# Large Induces Functional Glycans in an O-Mannosylation Dependent Manner and Targets GlcNAc Terminals on Alpha-Dystroglycan

**DOI:** 10.1371/journal.pone.0016866

**Published:** 2011-02-09

**Authors:** Yihong Hu, Zhi-fang Li, Xiaohua Wu, Qilong Lu

**Affiliations:** Neurology Department, McColl-Lockwood Laboratory for Muscular Dystrophy Research, Cannon Research Center, Carolinas Medical Center, Charlotte, North Carolina, United States of America; Universidade de São Paulo, Brazil

## Abstract

Alpha-dystroglycan (α-DG) is a ubiquitously expressed receptor for extracellular matrix proteins and some viruses, and plays a pivotal role in a number of pathological events, including cancer progression, muscular dystrophies, and viral infection. The O-glycans on α-DG are essential for its ligand binding, but the biosynthesis of the functional O-glycans remains obscure. The fact that transient overexpression of LARGE, a putative glycosyltransferase, up-regulates the functional glycans on α-DG to mediate its ligand binding implied that overexpression of LARGE may be a novel strategy to treat disorders with hypoglycosylation of α-DG. In this study, we focus on the effects of stable overexpression of Large on α-DG glycosylation in Chinese hamster ovary (CHO) cell and its glycosylation deficient mutants. Surprisingly, stable overexpression of Large in an O-mannosylation null deficient Lec15.2 CHO cells failed to induce the functional glycans on α-DG. Introducing the wild-type DPM2 cDNA, the deficient gene in the Lec15.2 cells, fully restored the Large-induced functional glycosylation, suggesting that Large induces the functional glycans in a DPM2/O-mannosylation dependent manner. Furthermore, stable overexpression of Large can effectively induce functional glycans on N-linked glycans in the Lec8 cells and ldlD cells growing in Gal deficient media, in both of which circumstances galactosylation are deficient. In addition, supplement of Gal to the ldlD cell culture media significantly reduces the amount of functional glycans induced by Large, suggested that galactosylation suppresses Large to induce the functional glycans. Thus our results revealed a mechanism by which Large competes with galactosyltransferase to target GlcNAc terminals to induce the functional glycans on α-DG.

## Introduction

Alpha-dystroglycan (α-DG), a highly glycosylated plasma membrane-associated protein, was originally isolated from brain and skeletal muscle [1], [2]. It is encoded by *DAG1* gene and expressed ubiquitously [3]. The *DAG1* gene is translated as a single polypeptide which is post-translationally cleaved into two subunits: α-DG and β-DG. The two subunits associate non-covalently as the key components of the dystrophin glycoprotein complex (DGC) [4]. Alpha-DG associates with extracellular matrix (ECM) proteins, while the transmembrane β-DG interacts with the sub-membrane dystrophin or utrophin, which is, in turn, linked to actin-based cytoskeleton. Proper glycosylation of α-DG is essential for its binding to the ECM proteins such as agrin, laminins, neurexin, and perlecan. The linkage between ECM and the cytoskeleton through DGC is critical for the membrane integrity and functions of skeletal muscles. Alpha-DG consists of three distinctive domains, an N-terminal globular domain, a central mucin domain, and a C-terminal globular domain. The mucin domain (317–488aa) has a cluster of 50 Ser/Thr residues, which are potential sites for O-glycosylation. Removing the O-glycans on α-DG abolishes its ligand binding, indicating that the O-glycans are essential for the activity [4], although the exact structure of the O-glycans mediating its ligand binding remains largely unknown. The importance of the O-glycans on α-DG has been illustrated by the discoveries that the mutations in known and putative glycosyl-transferase genes such as *POMT1/2, POMGnT1, LARGE, Fukutin,* and *Fukutin-related protein (FKRP)* cause aberrant O-glycosylation of α-DG and result in various muscular dystrophies with a wide spectrum of clinical manifestations (termed as dystroglycanopathies) [5], [6], [7], [8], [9], [10]. The hallmark of these diseases is the hypoglycosylation of α-DG and reduced binding of the α-DG to laminin. However, among these genes, only POMT1/2 and POMGnT1 have been demonstrated to have the glycosyltransferase activities in the protein O-mannosylation pathway [6], [11]. The roles of LARGE, Fukutin, and FKRP in glycosylation of α-DG remain to be defined.

Human LARGE was originally identified as a tumor related gene with deletion in meningioma [12]. It is a type II transmembrane glycoprotein with 756 amino acids, residing predominantly in the Golgi apparatus [13]. Its N-terminal and C-terminal domains have sequence similarities to bacterial α-glycosyltransferase and mammalian β-1,3-*N*-acetylglucosaminyl-transferase, respectively. Despite the fact that glycosyltransferase activity of the LARGE gene has not been demonstrated, accumulating evidence suggested that LARGE plays a critical role in biosynthesis of the functional glycans of α-DG, which can be detected by immuno-staining with the IIH6 and VIA4 monoclonal antibodies and laminin binding assays. Significantly, aberrant glycosylation of α-DG is frequently associated with a variety of tumors, which have LARGE expression silenced [14], [15], [16], [17]. Interestingly, Campbell and colleagues reported that transient overexpression of LARGE can functionally bypass distinct genetic defects in myoblasts derived from patients with mutations in *FCMD, POMT1,* and *POMTGnT1* [18]. Furthermore, previous studies also reported that transient expression of LARGE induced abundant functional glycans in the B421 and Lec15.2 cells with O-mannosylation defects, suggesting that transient overexpressing LARGE may process non O-mannosyl glycans to induce the functional glycans on α-DG [19], [20], [21], [22]. However, whether Large-induced functional glycosylation of α-DG is O-mannosylation dependent in physiological conditions remains unknown. Overexpressing LARGE can bypass hypoglycosylation of α-DG caused by non LARGE defects, suggested that overexpression of LARGE could be a potential therapeutic strategy for patients with hypoglycosylation of α-DG. However the detailed mechanism by which LARGE induces the functional glycans remains to be elucidated.

In the present study, we have utilized CHO cells and its glycosylation deficient mutants: Lec1, Lec2, Lec8, Lec15, and ldlD cells [23] and established an array of the cell lines with stable overexpression of mouse Large. With the O-mannosylation null deficient Lec15.2 cells, we demonstrated that stable overexpression of Large induces the functional glycans in a dolichol phosphate-mannose biosynthesis regulatory protein 2 (DPM2)**/**O-mannosylation dependent manner, thus confirming for the first time that Large is involved in modification of O-mannosyl glycans on α-DG. Quantitative analysis of the glycans on α-DG in the Lec8 and ldlD cells expressing Large revealed that Large can effectively induce the functional glycans on N-linked glycans. In addition, supplement of Gal to the conditioned media of ldlD-Large cell culture significantly reduced the amount of functional glycans, suggesting that galactosylation suppresses Large to generate the functional glycans. Our results revealed a mechanism by which Large competes with galactosytransferases to target GlcNAc terminals to generate the functional glycans on α-DG.

## Results

### Stable overexpression of Large induces the functional glycans independent of Gal, sialic acids, and complex N-linked glycans

**Figure 1:**
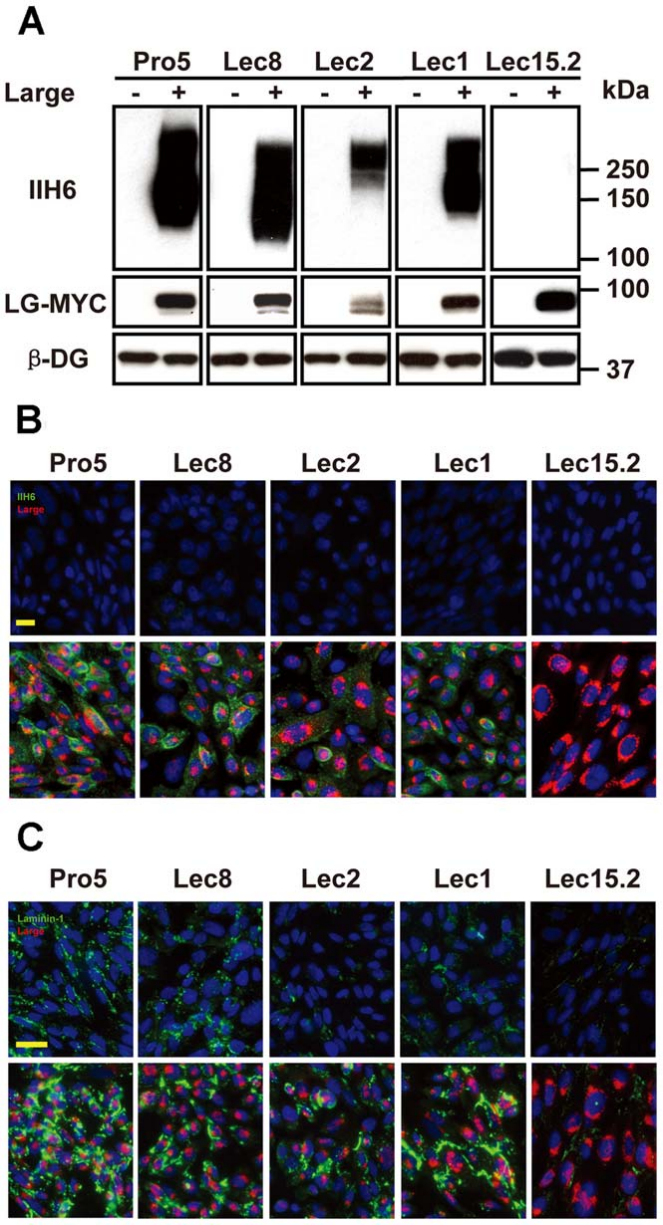
Stable overexpressing Large induces functional glycans in CHO cells. (A) Equal amount of lysates (60 µg proteins each lane) extracted from the cells stably expressing Large as indicated. The immuno-blotting procedure was described in methods section. The glycan antibody IIH6 and anti-MYC antibody (9E10) were used to detect the functional glycans and Large–MYC, respectively. In addition, an anti-β-DG (MANDAG2) antibody was used for detecting dystroglycan as the loading control. (B) The cells as indicated were seeded in 96-well plates and immuno-fluorescent staining was performed as described in the methods section. The images in top panel present the negative controls, while the images of bottom panel present the Large positive cells. A rabbit polyclonal anti-MYC antibody and the IIH6 antibody were used to stain the Large-MYC and glycosylated α-DG, respectively. The cells were also stained with DAPI to visualize the nucleus. The bar is 50 µm. (C) The cells as indicated were seeded in 96-well plate for 24 hour growth. The protocol of laminin clustering assay is described in the methods section. The laminins-DyLight488 was added to the cell culture for 6 hours incubation. A rabbit polyclonal anti-MYC antibody was used to stain Large-MYC protein followed by staining with secondary anti-rabbit antibodies conjugated with Alex594. In addition, DAPI was added to each well to stain the DNA for cells counting. The images were captured with a fluorescent microscope as described in methods section. The bar is 50 µm.

**Figure 2:**
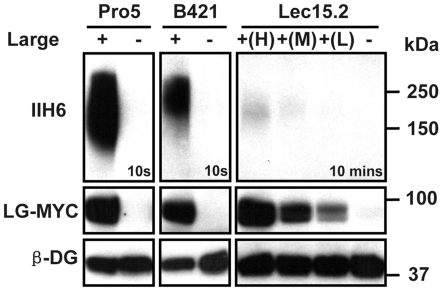
Stable overexpression levels of Large on glycosylation of α-DG in the Lec15 cells. Pro-5, B421 clones overexpressing (+) or without (−) Large-MYC or Lec15.2 cells overexpressing Large-MYC at high +(H), modest +(M) low +(L) or without Large-MYC (−) were tested by immuno-blot assay. The blot of the Lec15.2 samples had 10 mins exposure, while all other blot had about 10 seconds' exposure time.

Previous reports on Large function were all carried out with transient overexpression, which often have extremely high but unsustainable levels of Large expression [19], [20], [21], [22]. To investigate the effect of stable overexpression of Large on specific glycans, we have established an array of glycosylation deficient CHO cell lines (Pro5, Lec1, Lec2, and Lec8), in which mouse Large tagged with a MYC epitope was stably expressed and termed as Pro5-LG, Lec1-LG, Lec2-LG, and Lec8-LG, respectively. The Lec2 and Lec8 cells are deficient in the Golgi CMP-sialic acid transporter and UDP-Gal transporter, respectively [23]. Consistent with previous reports with transient Large overexpression, stable overexpression of Large in both cells induced abundant functional glycans on α-DG as determined by the IIH6 monoclonal antibody, which recognizes the epitope(s) overlapping with laminin binding glycans on α-DG. Glycans on α-DG recognized by both the antibodies and laminin binding assay have been widely referred to as the functional glycans [24]. Similarly, the functional glycans were produced abundantly in the Lec1-LG cells deficient in enzyme MgalT1, which is essential for complex N-glycan formation on proteins [23]. The biological functions of the Large-induced functional glycans on α-DG in these cells were further supported by their cell surface localization examined by immuno-fluorescent microscopy with the IIH6 antibody (Fig. 1B) and the laminin cluster assay (Fig. 1C). These results are consistent with previous observation using transient expression methodology (Fig. 1, 2), suggested that stable overexpression of Large induced the functional glycans independent to Gal, sialic acids, and complex N-linked glycans.

### Stable overexpression of Large induces the functional glycans in a DPM2 dependent manner

Large has been thought to modify O-mannosyl glycans to produce the functional glycans on α-DG to mediate its ligand binding. However, so far there has been no direct experimental evidence to support the hypothesis. To investigate the role of Large in o-mannosylation of α-DG, we established a stable cell line with Lec15.2 cells, in which Large was stably expressed. The Lec15.2 cell is deficient in DPM2 that plays an essential role in the complex of DPMs to produce dolichol-phosphate-mannose (Dol-P-Man). The Dol-P-Man is the substrate for enzymatic complex of POMT1/2 to initiate O-mannosylation of α-DG by adding the first mannose to Ser/Thr residues of α-DG in the lumen of ER [11]. Surprisingly, stable overexpression of Large failed to induce significant amount of the functional glycans in the Lec15.2 cells. The amount of the functional glycans was undetectable in the lysates from the Lec15.2-LG cells with the IIH6 antibodies, while its Large expression level is compatible to that in the other CHO-LG cell lines (Fig. 1A). The lack of the functional glycans on the Lec15.2-LG cells was further confirmed by the immuno-staining and laminin clustering assay (Fig. 1B, C). The results are apparently in contradiction to the previous reports in which several groups showed that transient overexpression of Large induced abundant functional glycans in either the B421 or LG 15.2 cells which are both deficient in the *DPM2* gene [19], [20], [21], [22]. The Lec15.2-LG cells used in this study were selected from a large population of Lec15.2 cells. One possibility is that the selected Lec15.2-LG clones might have acquired additional genetic defects which may compromise the activity of Large. To address this issue, we independently repeated Large transfection of the Lec15.2 cells (3 times) and screened 1,000 clones overexpressing Large by indirect immuno-fluorescent microscopy. About 10% of transferant cells were capable of producing detectable IIH6 reactive glycans on the cell surface within 4-6 weeks after transfection. However, majority of the Lec15.2-LG clones gradually lost the capacity to induce the function glycans after 20 passages in continuing cell culture for 4 months (Fig. 2) despite the fact that the levels of Large expression are compatible to that in other tested CHO-LG cell lines (Fig.1 and 2). Four Lec15.2-LG clones retained the capacity of inducing functional glycans, but at much lower levels when compared to the other CHO-LG cells including Lec2-LG, Lec8-LG, and B421-LG cells. Three of the 4 IIH6 positive clones (Lec15.2-LG) were further examined by Western blotting for the amount of functional glycans and levels of Large expression together with Pro5-LG, B421-LG, and negative controls (Fig. 2). For comparison, the B421 cells were also transfected with the same Large expression construct and a number of clones were selected. In contrast to the Lec15.2-LG clones, all the B421-LG clones stably overexpressing Large maintained production of abundant functional glycans after 4 months culture (the same period for Lec15.2 clones), although the amount of functional glycans were about 50% of that in Pro5-LG cells (Fig. 2). The expression levels of Large in these B421-LG cells were similar to other CHO-LG stable cell lines including the Lec15.2-LG clones by the Western blotting assay. The consistent results between independent Large transfection experiments suggested that additional genetic defects are unlikely the reason responsible for loss of Large activity in the Lec15.2 cells.

**Figure 3:**
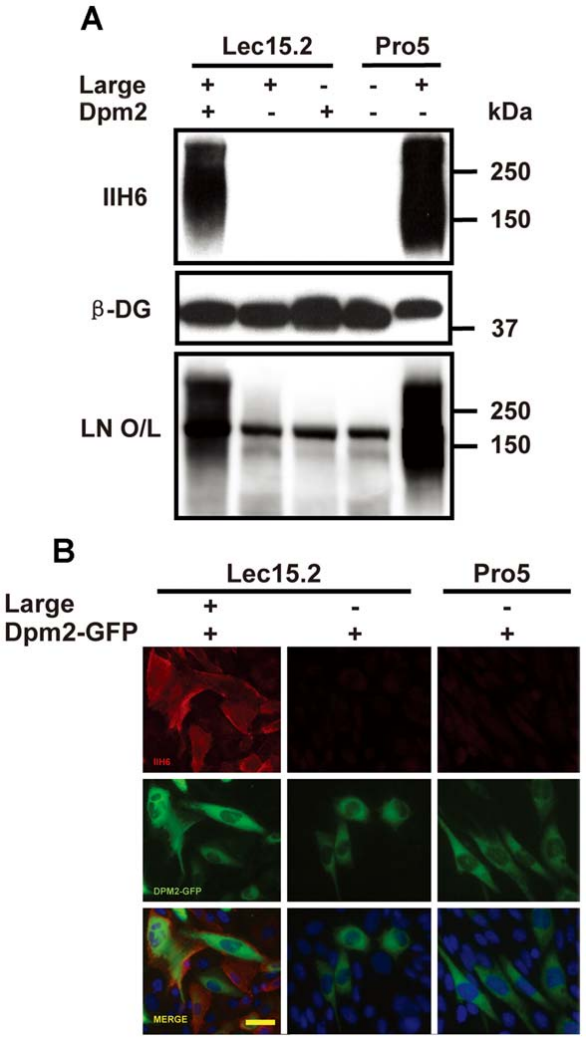
Overexpressing DPM2 restores the Large function in the Lec15.2 cells. (A) Transient transfection was conducted to introduce mouse *DPM2* cDNA into the Lec 15.2-LG cells. 48 hours after transfection, the cell lysates were harvested and immuno-blotting assay and laminins overlay assay were conducted as described above. (B) A transient transfection was conducted to introduce DPM-GFP cDNA into the cells in 96-well plate. 48 hours the after transfection, cells were fixed and an indirect immuno-fluorescent staining with IIH6 antibody was conducted with the protocol as described in the methods section. The images were captured with a fluorescent microscope as described in methods section. The bar is 50 µm.

To further address whether the deficiency of DPM2 is responsible for the loss of the Large function in the Lec15.2 cells, we conducted complementation experiments by introducing a wild-type mouse *DPM2* cDNA into the Lec15.2-LG cells. In the experiments, the Lec15.2-LG cells were transfected with the *DPM2* mammalian expression construct, and then examined by Western blot and laminin overlay assays (Fig. 3A). The DPM2 transfection restored Large function in the Lec15.2-LG cells, while DPM2 expression alone did not produce detectable the functional glycans. Furthermore, we also overexpressed DPM2-GFP in the Lec15.2-LG cells and examined functional glycans on the cell surface. Indeed, the expression of DPM2-GFP restored the Large function to produce the functional glycans in the Lec15.2-LG cells, while expression of DPM2-GFP alone failed to do so in either the Lec15.2 or the Pro5 cells (Fig. 3B). The results suggested that stable overexpression of Large induces the functional O-glycans on α-DG to mediate laminin binding in a DPM2/O-mannosylation dependent manner.

### Stable overexpression of Large can effectively modify N-linked glycans in the absence of galactosylation

**Figure 4:**
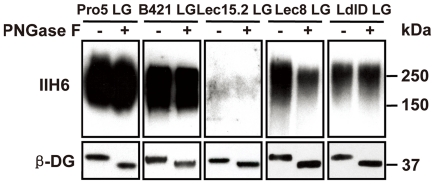
Effects of Large-induced pathway on N-linked glycans. (A) All cells were grown in the completed media as described in the methods section and the lysates harvested from the indicated cells with stably expressing LARGE were treated with pNGase F for 4 hours, while it is absent in the negative controls reactions as described in the methods section. IIH6 antibody was used to determine the LARGE induced glycans, and the anti-β-DG antibody was used to determine the β-DG proteins as described above. The blot of the Lec15.2 samples with the IIH6 antibody had 30 mins exposure, while all other blots had about 10 seconds' exposure time.

It has been reported that transient overexpression of Large modifies complex *N*-glycans on α-DG, particularly, in the Lec8 cells [19]. To investigate whether stable overexpression of Large has effect on N-linked glycans of α-DG as well, we used PNGase F to remove the N-linked glycans on α-DG from the Pro5-LG, B421-LG, Lec15.2-LG, Lec8-LG and ldlD-LG cells growing in the complete media (Fig. 4). The reduction of IIH6 staining signal in Pro5-LG, B421-LG, and Lec8-LG represents the amount of functional glycans on N-linked glycans and the remaining functional glycans would be on O-glycans. In the Lec15.2-LG cells, the remaining functional O-glycans would be on mucin type of O-glycans only since the cells are lack of O-mannosyl glycans due to missing DPM2 activity. Interestingly, removing N-linked glycans did not reduce significant amount of functional glycans on α-DG in all tested stable cell lines, except the Lec8-LG cells. The remaining functional glycans after PNGase F digestion was reduced to about 50% of that of untreated samples from Lec8-LG cells, suggesting that stable overexpression of Large only modifies N-linked glycans on α-DG to induce the functional glycans effectively in the galactosylation deficient Lec8 cells.

### GalNAc enhances, whereas Gal and Gal/GalNAc inhibit production of Large-induced functional glycans in ldlD-LG cells

**Figure 5:**
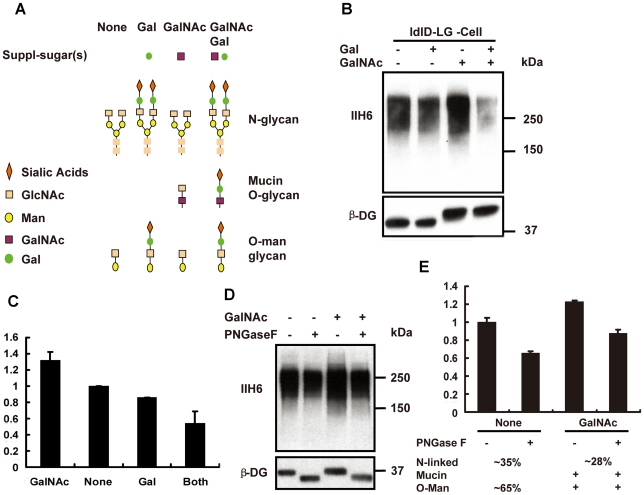
LARGE modifies both mucin type O-glycans and N-linked glycans on α-DG in ldlD cells while without galactosylation. (A) Predicted structures of the glycans in N-linked, Mucin and O-mannosyl pathway in the ldlD cells. (B)The ldlD-LG cells were maintained in F12 nutrition mix media with 3% lipoprotein deficient bovine serum for more than 48 hours prior to the experiment. The cells were seeded into 6-well plates one day before addition of indicated sugars (Gal at 10 µM and GalNAc at 200 µM). After being treated with the sugar(s) for 24 hour, the cell lysates were harvested and equal amount of the lysates were loaded. An immuno-blotting assay was performed to detect the functional glycans and β-DG with the IIH6 and β-DG antibody, respectively. (C) The quantitative data of the expression levels of the functional glycans were obtained with AlphaImage AIC software based on densitometers followed the manufacture instructions. The IIH6 expression levels were normalized with the expression levels of β-DG (N = 3). (D) The lysates harvested from the ldlD-LG cells growing in the conditions as indicated and the experimental procedure is the same as described in (Fig. 4A). (E) Quantitative analysis of the data of (D) (N = 3).

The structure of Large-induced functional glycan is unknown; and there is no method available to selectively remove O-mannosyl or mucin-type of O-glycans. Thus it is difficult to study the effects of Large on O-mannosyl and mucin-type O-glycans, separately. To circumvent this hurdle, we took advantage of the reversible glycosylation deficient CHO cells, ldlD cells, which are deficient in UDP-galactose 4′-epimersase (GALE) and produce neither UDP-GalNAc nor UDP-Gal resulting in pleiotropic glycosylation pathway defects [25]. Supplement of GalNAc and Gal to the cell culture media can restore the glycosylation pathways due to a salvage pathway in the CHO cells (Fig. 5A) [25]. Thus the mucin-type O-glycan biosynthesis could be manipulated by supplementing the sugar(s) to the cell culture media. In the experiments, the stable ldlD-LG cells were grown in the media with 3% lipoprotein deficient serum for 48 hours to deplete the intracellular UDP-GalNAc and UDP-Gal pools [25]. Under this culture condition the ldlD cells do not produce detectable mucin-type O-glycans by radioisotope labeling methodology [25]. We examined the amount of the functional glycans produced in the ldlD-LG cells growing in the cell culture media with different sugar supplements. Strikingly, the ldlD-LG cells produced the least amount functional glycans when grown in the medium supplement with both Gal and GalNAc, despite the glycosylation pathways were restored and evidenced by the β-DG immuno-blotting (Fig. 5). In contrast, the cells produced the most abundant functional glycans when supplemented with GalNAc alone. The comparison of the amount of the functional glycans produced in the ldlD-LG cells growing in different culture media [GalNAc (2.4) > non sugars (1.8)> Gal (1.6)>GalNAc and Gal (1)] is shown in Fig. 5B,C. To further analyze the effects of Large on N-linked and O-linked glycans quantitatively in the ldlD-LG cells, we removed the N-glycans from the α-DG proteins with PNGase F, which were harvested from the conditioned culture media with or without GalNAc (Fig. 5D, E). The results showed that without supplement of Gal and GalNAc, ∼35% of the Large-induced functional glycans were on N-linked glycans (digested portion) and ∼65% were on O-mannosyl glycans (remaining portion), since the mucin-type of the O-linked glycans are expected to be minimal. Supplement of GalNAc alone increases the total functional glycans by about ∼28% comparing with that in the no sugar addition and about 25% of the functional glycans are on N-linked glycans (Fig. 5D,E).

## Discussion

In this study, we demonstrated that Large, a putative glycosyltransferase, induces the functional glycans on α-DG to mediate laminin binding in a DPM2/O-mannosylation dependent manner. Stable expression of Large failed to induce the functional glycosylation on α-DG, but it can be restored by expressing DPM2 in the Lec15.2-LG cells. Thus our results confirmed for the first time that Large is involved in the biosynthesis of the O-mannosyl glycans suggested by the results from clinical and mouse genetic studies. This result is also in-line with the recent report that the functional glycans may be linked to the first mannosyl residue of the O-mannosyl glycans on mucin domain [26]. It was curious to learn that the discrepancy between the results of the effects of stable expressing Large on α-DG glycosylation in the two Lec15 clones, B421 and LG15.2 cells. It can be explained that the B421 cells harbor the point mutation form of *DPM2* which may allow the cells to produce sufficient amount of Dol-P-Man to support the function of stable expressing Large to produce abundant functional glycans. But the Lec15.2 cells produce neither *DPM2* message RNA nor Dol-P-Man that is essential substrate for POMT1/2 complex to initiate the biosynthesis of O-mannosyl glycans in the ER lumen, thus resulting in loss of the Large activity in the Lec15.2 cells. On the other hand, when transient overexpression at extremely high level Large can produce the functional glycosylation of α-DG in the Lec15.2 cells (data not shown), suggested that Large can induce the functional glycans on N-linked and mucin O-glycans to bypass defects in the early steps of O-mannosylation pathway. But it can not be sustained.

Our results obtained from the sugar addition experiments with ldlD-LG cells clearly suggested that Gal inhibits the ability of Large to induce the functional glycans on the N-linked and mucin-type O-glycans. The mucin-type O-glycans consist of four components: Sia, Gal, GlcNAc, and GalNAc. Sia, Gal, and GalNAc are not the essential components of the functional glycans and addition of Gal or both Gal and GalNAc together even inhibited the functional glycosylation induced by Large. Thus only the GlcNAc could be the Large's target on mucin-type of O-glycans. This also explains that Large induces the functional glycosylation on N-glycans effectively in galactosylation deficient cells (Lec8) since the GlcNAc terminals are enriched on the N-glycans in galactosylation defective condition. Our results suggested that Large-pathway targets GlcNAc terminals to generate the functional glycans in various glycans. A recent study also suggested that β-1,3-*N*-acetylglucosaminyl-transferase is essential for functional glycosylation of α-DG in a prostate cancer cell line, supporting the view that GlcNAc terminals may be the target of Large-pathway[22].

Surprisingly, addition of GalNAc alone to the conditioned media of ldlD-LG cell culture remarkably enhances the Large-induced functional glycans on α-DG. In contrast, addition of both Gal and GalNAc to the ldlD-LG cell culture media resulted in producing much less functional glycans in the cells despite all glycosylation pathways are supposed to be restored in this condition. The phenomenon may be explained as follow. When the ldlD cells grown in the media without Gal/GalNAc, Large-pathway targets the GlcNAc terminals on O-linked and N-linked glycans to produce abundant functional glycans. Supplement of Gal alone to the culture media allows the cells to add Gal to the GlcNAc terminals on O- and N-linked glycans and results in inhibiting Large-pathway to target GlcNAc terminals to generate the functional glycans. While supplement of GalNAc alone to the cell culture the ldlD-LG cells add more GalNAc to the abundant Ser/Thr (up to 50) residues on the mucin domain to initiate the biosynthesis of mucin O-glycans. Sequentially, the GalNAc residues would be modified by endogenous βGlcNAc-transferases (βGn-Ts) to produce more GlcNAc terminals, since Gal is not available. Thus, Large modifies the abundant GlcNAc terminals to produce the most amount of the functional glycans among the cell culture conditions. Finally, while supplement of both Gal and GalNAc to the cell culture media, βGal-transferases (βGal-Ts) would modify GlcNAc terminals on all types of glycans, thus preventing Large to modify GlcNAc terminals to produced the functional glycans. Our data also suggested that fully restoration of mucin type of O-glycosylation with supplement of both Gal and GalNAc may compete with the functional O-mannosylation induced by Large. This is supported by recent report that the O-GalNAcylation and O-Mannosylation pathways are competing for the same sites of modification on α-DG[27]. Thus Large produced the least amount of the functional glycans among the cell culture conditions (Fig.5). The difference between the functional glycans biosynthesis in the different glycosylation conditions is unlikely due to protein degradation or other nonspecific effects caused by glycosylation defects. Since the functional glycans biosynthesis is suppressed in the better glycosylation conditions rather than in the worse conditions (Fig. 5).

**Figure 6:**
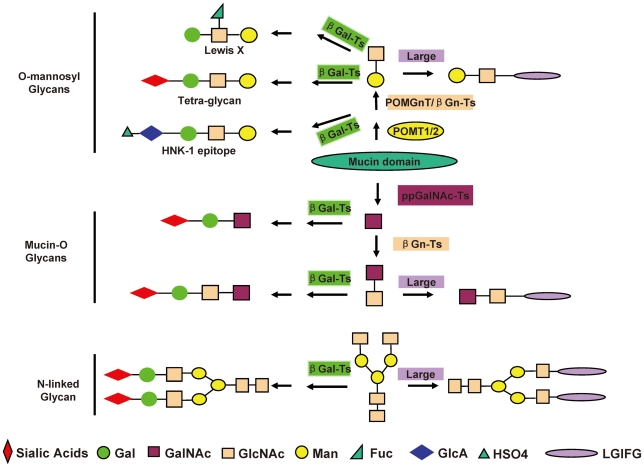
Illustration of the role of Large in the glycosylation of α-DG. LGIFG: Large-induced functional glycans.

To date, four O-glycans structures on α-DG have been reported: NeuAcα2-3Galβ1-4GlcNAcβ1-2Man (the tetra-glycan) from bovine brain and rabbit skeleton muscles [28], [29]. Galβ1-4(Fucα1-3)GlcNAcβ1-2Man (Lewis^X^ structure) from sheep brain [30], HSO3-3GlcAβ1-4GlcNAcβ1-2Man (HNK1 epitope) from rat brain [31], [32], and Galβ1-3GalNAc (Core-1) from myotubes [33]. The diversity of the O-glycan structures on α-DG allows α-DG to play distinct roles in various tissues to mediate the interactions with its variety of ligands. For example, HNK-1 epitope containing O-glycan mediates α-DG binding to laminins 10/11 in brain [32], while its core-1 structure of mucin-type glycan on α-DG mediates laminin-induced acetylcholine receptor clustering but not laminin binding activity in myotubes [33]. Our results suggested that the NeuAcα2-3Galβ1-4GlcNAcβ1-2Man glycan can not be the Large-induced functional glycans mediating laminin binding. Considering the N- and C-terminal domains of LARGE is related to bacterial α-glycosyltransferase and β-1,3-*N*-acetylglucosaminyl-transferase, respectively. In addition, Large itself is able to bind to α-DG. It is likely that Large function as glycosyltransferase modifying the GlcNAc to generate the functional glycans. Although the structure of Large-induced functional glycan remains to be determined, Our results nevertheless provided insights into the glycosylation of α-DG, which are illustrated in the Fig.6. 1) Large predominantly modifies O-mannosyl glycans to produce the functional glycans at stable overexpression levels in CHO cells, implicating that so does the endogenous Large. 2) Over expression of Large competes to modify GlcNAc terminals with galactosylation to generate the functional glycans on both O-linked and N-glycans, suggesting that suppressing galactosylation to produce more GlcNAc terminals on α-DG would significantly enhance Large function to produce the functional glycans, which may be a potential way to enhance the biosynthesis of the functional glycans on α-DG.

## Materials and Methods

### Antibodies and Laminins

Mouse anti-glycosylated α-DG mAb IIH6 (Upstate Biotechnology, Inc.) was used at 1∶200 and 1∶1000 dilutions for Immuno-fluorescent staining and Immuno-blotting assay, respectively. Anti-β-DG MAb 43DAG1/8D5 (hybridoma conditioned medium) from DHSB was used at 1∶200 dilution. Mouse anti-MYC mAb 9E10 (DHSB) was used at 1∶500 dilution. Rabbit anti-GM130 antibody (Stressgen) was used at 1∶500 dilution. Rabbit anti-laminin-1 polyclonal antibody (Sigma) was used at 1∶400 and 1∶1500 dilution for Immuno-fluorescent staining and immuno-blotting assay, respectively. Anti-GFP mAb (JL-8) (Clontech) was used at 1∶100 for immuno-precipitation and 1∶5000 for immuno-blotting assay. Laminins (laminin from Engelbreth-Holm-Swarm murine sarcoma basement membrane, Sigma) was used at dilution 5 µg/ml for laminins overlay assay. Horseradish peroxidase-conjugated anti-mouse, anti-rabbit, and anti goat secondary antibodies (Sigma) were used at 1∶5000–1∶10,000 dilutions. Alexa-488, 594 conjugated secondary antibodies (Invitrogen) were used at 1∶200–1∶500 dilutions.

### Expression Constructs

The mouse LARGE construct in pcDNA3.1/Myc-His(-)B vector was provided by Pamela Stanley [19]. Mouse DPM2 constructs in pCMV-Kan/Neo vector with or without GFP tag were purchased from Origene. The construct for human α-dystroglycan tagged with human Ig FC-fragment (HIG) was generated by cloning the a-DG cDNA fragment into the Pfuse-higG1-Fc vector (Invivogen). All constructs were confirmed by sequencing in Carolinas Medical Center molecular biology facility.

### Cell Culture, Transfection, and Stable Cell Lines

Pro5 and the glycosylation-deficient CHO mutants termed Lec1, Lec2, and Lec8 were purchased from ATCC [23]. The Lec15.2 and B421 cell lines were kindly provided by Mark Lehrman and Sharon Krag, respectively [34], [35]. These CHO cells were grown and maintained in F12 nutrition mix media with 10% fetal bovine serum (Invitrogen) in plates with 5% CO_2_ at 37°C, except that the Lec15 cells were grown at 34°C. For transfection, the cells were seeded in 6-wells plates, and were transfected using Fugene 6 reagents (Roche) followed the manufacturer's guidelines. For selecting stable cell lines expressing LARGE-MYC, the cells were cultured with G418 at 1.2 mg/ml (Invitrogen) 48 hours after transfection. The clones resistant to G418 were screened with laminin-dylight488. The positive clones binding to laminin-Dylight488 were picked and further confirmed with immuno-fluorescent staining with an anti-MYC monoclonal antibody.

### Electrophoresis of Cell Lysates and Immuno-blotting

Cell lysates were prepared from stable cell lines or transient transfectants 2 days after transfection. For each 10 cm plate, 1 ml of lysis buffer (1.5% Triton X-100 in PBS with protease inhibitor cocktail from ROCHE) was used to harvest the cells. The concentration of proteins in lysates was determined by Bio-Rad Dc protein assay. For electrophoresis, lysates (60 µg of protein) were boiled for 5 min in SDS-PAGE gels loading buffer. After electrophoresis in 4–12% gradient polyacrylamide gels (Invitrogen) at 168 V for 90–120 min, proteins were transferred to polyvinylidene difluoride membrane for 3 hours at 200 mA in transfer buffer containing 10% methanol. For immunoblotting, membranes were blocked in PBS with 10% fat free milk and incubated in the same solution with primary antibody at room temperature for 1 hour. After washing in PBS for 5X5 min, membrane was incubated in PBS/milk with horseradish peroxidase-conjugated secondary antibody for 1 hour at room temperature. After washing in PBS for 5X5 min, membrane was incubated in Super Signal West Pico™ chemiluminescence reagent (Pierce) and exposed to photographic film (Eastman Kodak Co.).

### Laminin Binding Assay with Cells

The cells were seeded in 96 well plates for overnight incubation. Then the cells were fixed and permeablized in 0.2% Triton X-100 in the laminin overlay buffer (LBB, 10 mM ethanolamine, 140 mM NaCl, 1 mM MgCl_2_ and 1 mM CaCl_2_, pH 7.4) for 8 min and blocked with 10% BSA for 1 hour at 37°C. Then 50 µl of 5 µg/ml laminins-DyLight-488 labeled in the LLB was incubated with the cells for 2 hours at 37°C. The plates were washed for 6 times with a micro-plate washer (BioTeck EXL405) and read with Infinite 500 microplate reader (TECAN). For staining the LARGE-MYC, an immuno-staining experiment was conducted with the anti-MYC polyclonal antibody.

### Laminin Clustering Assay

The cells were seeded in 96 well plates for overnight incubation. Then the cells were incubated with 50 µl of 5 µg/ml laminins-DyLight-488 labeled in LBB for 6 hours at 37°C in cell culture incubator. The cells were washed with LLB twice and fixed. The cells were washed twice again and 50 µl LLB per well were added, and plates will be ready for microplate reading or capturing images with fluorescent microscopy.

### Laminin Overlay Assay

The cell lysates were separated by SDS-PAGE gels and transfered to Nitrocellulose membranes. The membranes were blocked in 10% non-fat milk in LLB for 1 hour, and then incubated with 2.5 µg/ml laminin at 4°C overnight. After washing with LLB 5x5 min the laminin binding to α-DG was detected by standard Immunoblotting as describe above with a laminin polyclonal antibody (Sigma) followed by a goat anti-mouse IgG-HRP secondary antibody.

### Immuno-fluorescent Microscopy

The cells were fixed, permeablized, and then blocked with 10% BSA in PBS for 1 hour. The primary antibodies in PBS with 10% BSA were incubated with the cells for 1 hour at room temperature (22°C) followed by addition of secondary antibodies conjugated with Alexa-488/594. After washing the cells with PBS for 2 times, the DAPI was used to stain the cells for 15 mins and wash 3 times with PBS. The images were captured with an inverted Olympus fluorescent microscope.

### PNGase F digestion

The deglycosylation procedure was followed the instruction by manufacture (NEB, Ipswich, MA). 50 µg of total protein extracted from indicated cells were boiled for 10 min in 1x Glycoprotein Denaturing Buffer followed by incubation with 500 units of PNGase F at 37°C for 4 hours. Samples were analyzed by immuno-blotting assay as described above.
